# Supplementary material for: Introducing CAR-T Therapy in Kazakhstan: Establishing Academic-Scale Lentiviral Vector and CAR-T Cell Production
Source: Biomolecules. 2025 Aug 14;15(8):1166. doi: 10.3390/biom15081166 (PMC12385160; doi:10.3390/biom15081166)
Supplement: Supplementary file 1 [file biomolecules-15-01166-s001.zip › biomolecules-3773826-supplementary.pdf]

Supplementary material to

Introducing CAR-T Therapy in Kazakhstan: Establishing Academic-Scale Lentiviral Vector and CAR-T Cell Production

Viktoriya Keyer [1], Aitolkyn Kydyrbayeva [1], Tolganay Kulatay [1], Gulzat Zauatbayeva [1], Dmitrii Bazhenov [1], Bakytkali Ingirbay [1], Zhanar Shakhmanova [1], Maral Zhumabekova [1], Madina Ospanova [2], Alexandr V. Shustov [1,*]

[1] National Center for Biotechnology, Korgalzhin hwy 13/5, 010000, Astana, Kazakhstan

[2] Scientific-Production Center of Transfusiology, Zhanibek Khandar St., 10, 010000, Astana, Kazakhstan

***Correspondence**:

Alexandr V. Shustov, shustov@biocenter.kz , phone: +77024735305 (Watsup)

**V.K.**: keer@biocenter.kz	ORCID 0000-0001-8885-2387
**A.K.**: aitolkyn.yk@gmail.com	ORCID 0009-0003-2199-5756
**T.K.**: Kulatay@biocenter.kz	ORCID 0009-0004-5885-8963
**G.Z.**: zauatbaeva@biocenter.kz	ORCID 0000-0003-1514-9302
**D.B.**: bazhenov@biocenter.kz	ORCID 0000-0003-4532-7720
**B.I.**: ingirbay@biocenter.kz	ORCID 0000-0002-6915-8207
**Z.S.**: zhanar.shakhmanova@bk.ru	ORCID 0009-0000-0471-2388
**M.Zh.**: zhumabekova@biocenter.kz	ORCID 0009-0002-9532-6232
**M.O.**: sk_ospanova_me@mail.ru	ORCID 0000-0003-4305-4351
**A.V.S.**: shustov@biocenter.kz	ORCID 0000-0001-9880-9382

Table S1. Demographic data and diagnoses of patients who underwent leukapheresis [1]

Product #	Patient age/gender	Purpose of leukapheresis procedure	Disease
1	45/M	Stem cell harvesting	NHL (DLBCL)
2	33/M	WBC reduction	Hyperleukocytosis
3	48/M	Stem cell harvesting	Myelodysplastic syndrome
4	60/M	Stem cell harvesting	MM
5	47/M	Stem cell harvesting	CLL
6	31/M	Stem cell harvesting	AML
7	42/M	Stem cell harvesting	CLL
8	27/F	Stem cell harvesting	CLL
9	55/M	Stem cell harvesting	Erythroid leukemia
10	33/M	Stem cell harvesting	AML
11	48/F	Stem cell harvesting	NHL
12	21/F	Stem cell harvesting	CLL

[1] M: male; F: female; AML: acute myeloid leukemia; CLL: chronic lymphocytic leukemia; DLBCL: diffuse large B-cell lymphoma; MM: multiple myeloma; NHL: Non-Hodgkin lymphoma.

Figure S1. Gating strategy for flow cytometry analysis of immune cell composition in the starting cell material

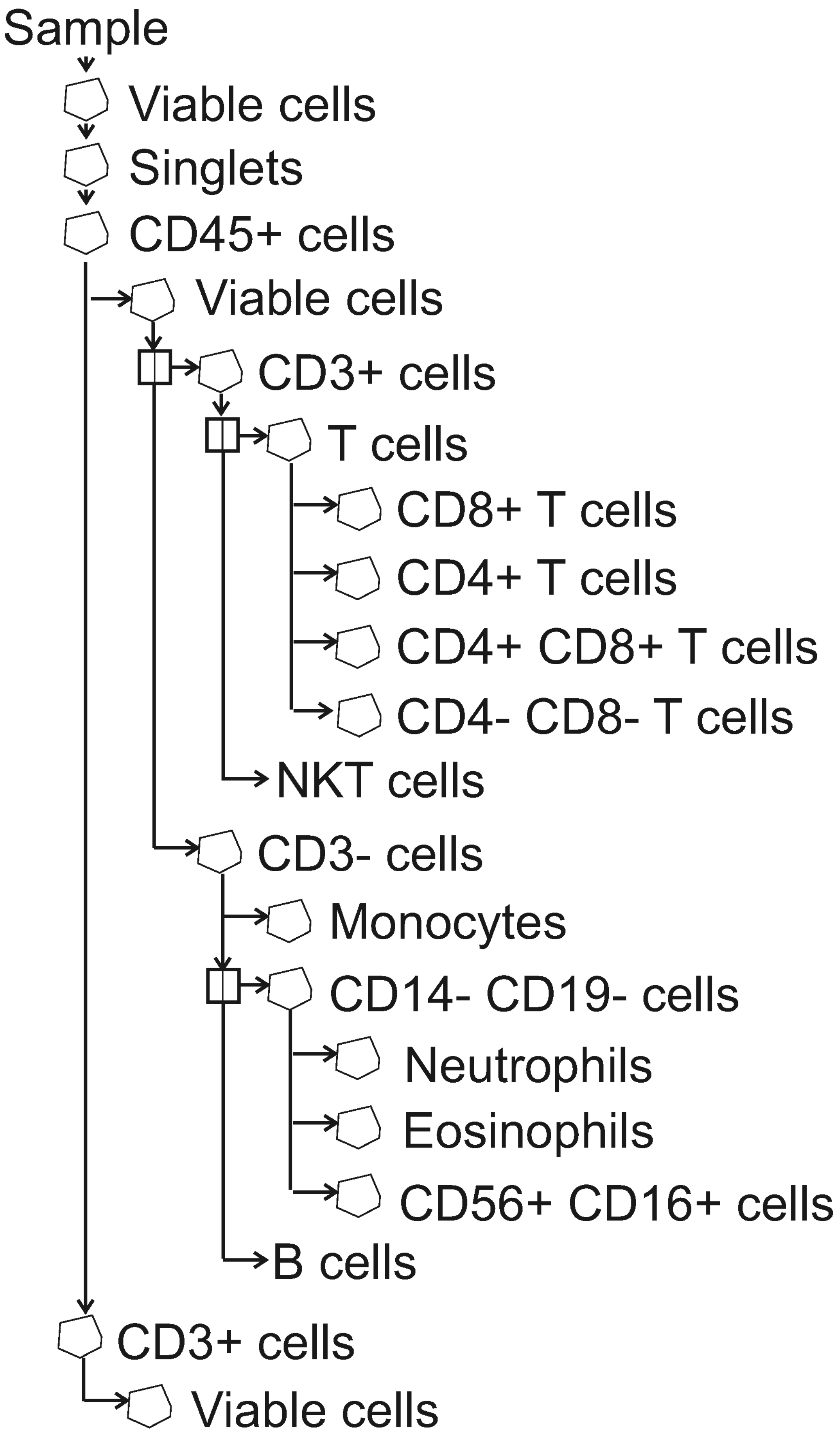

Figure S2. Gating strategy for flow cytometry analysis of the CAR-T cell product

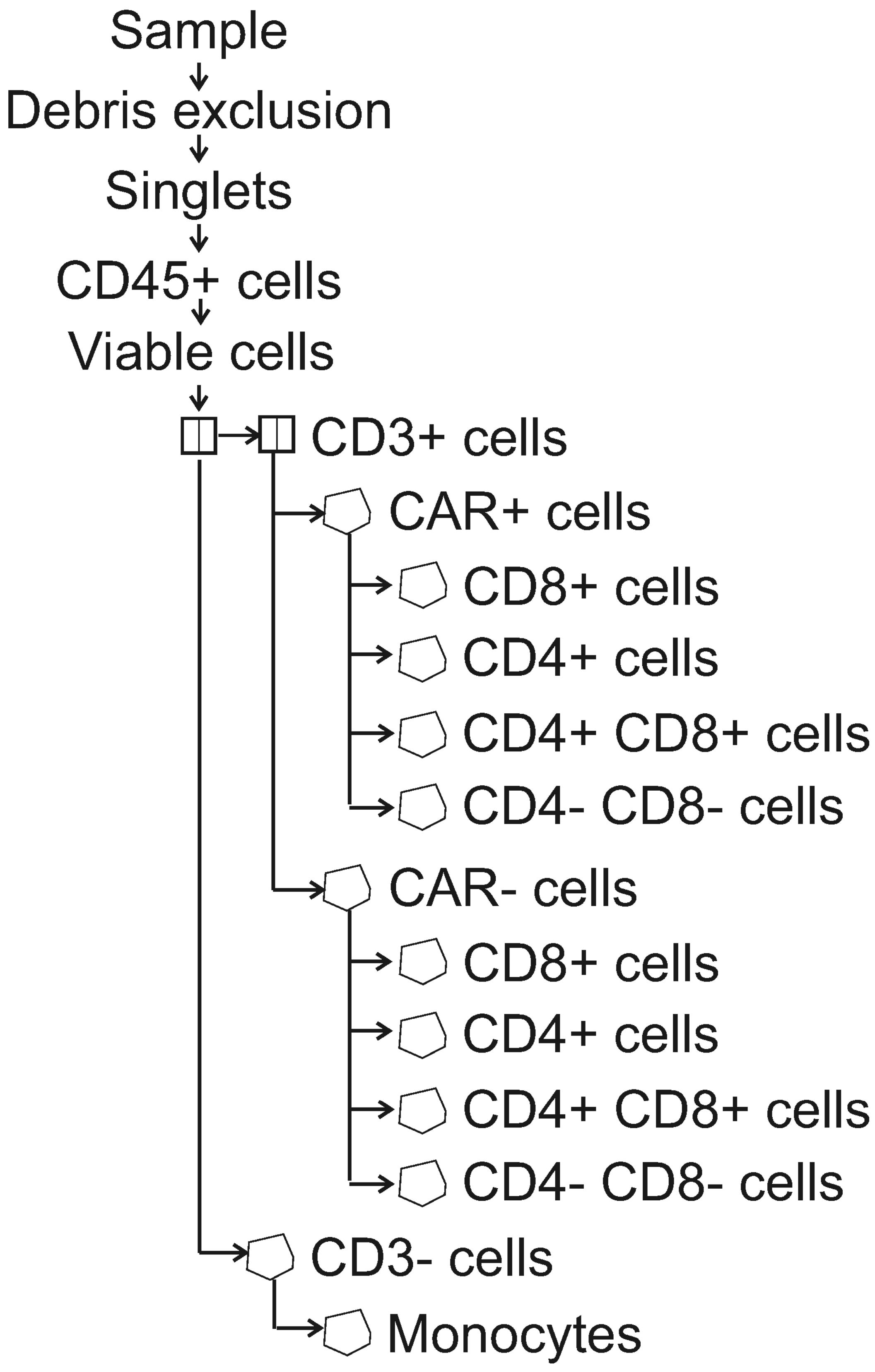

Figure S3. Gating strategy for immunophenotyping T cell subsets

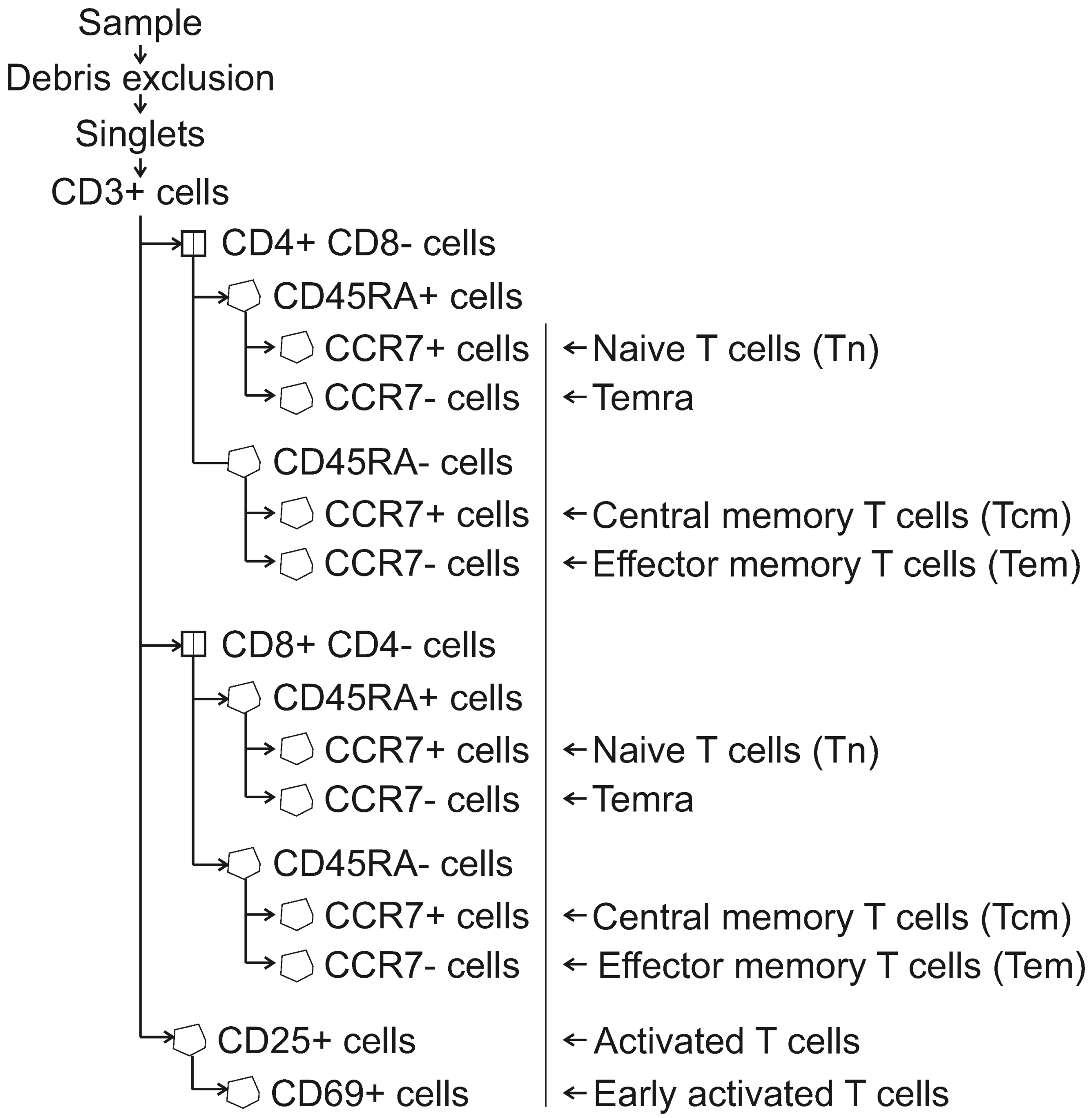

Figure S4. Gating strategy for quantification of PKH67-labeled B cells in co-cultures

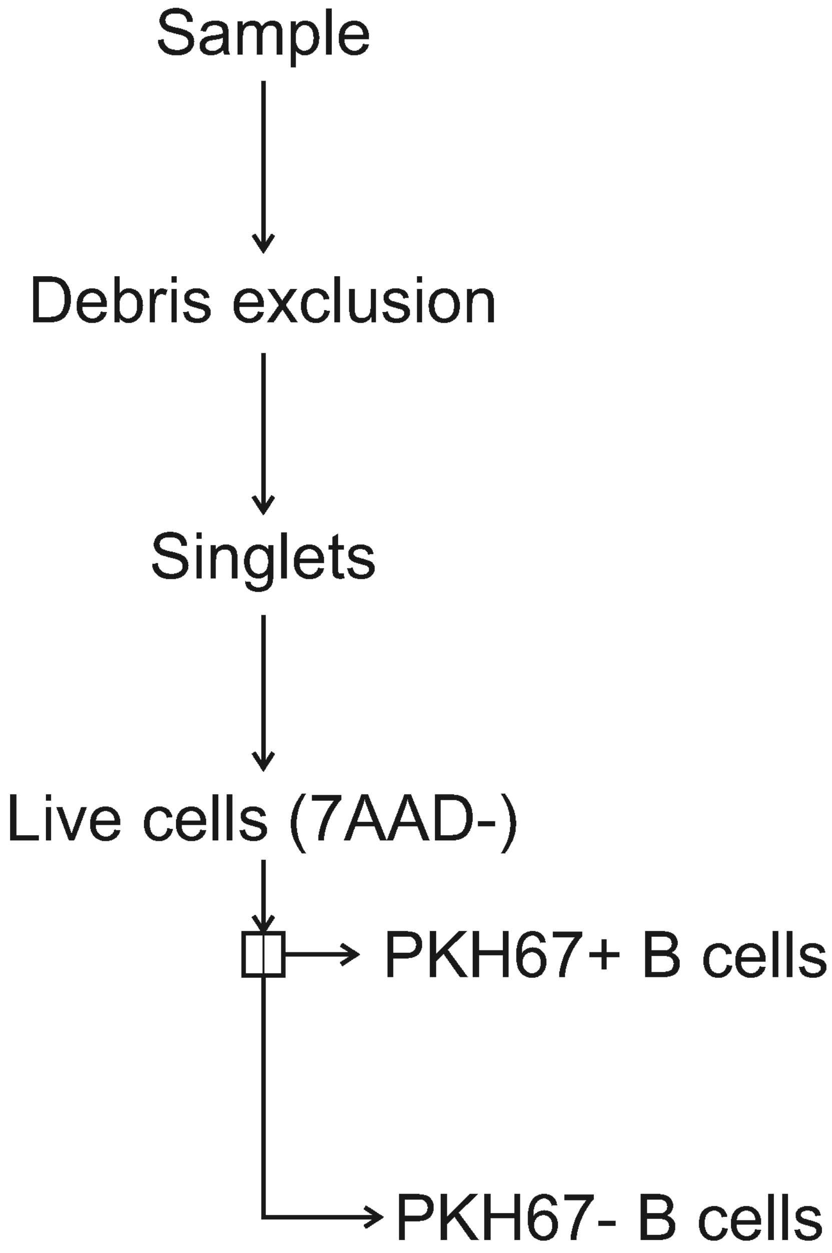

Figure S5. Representative analysis of immune cell composition in apheresis product

a

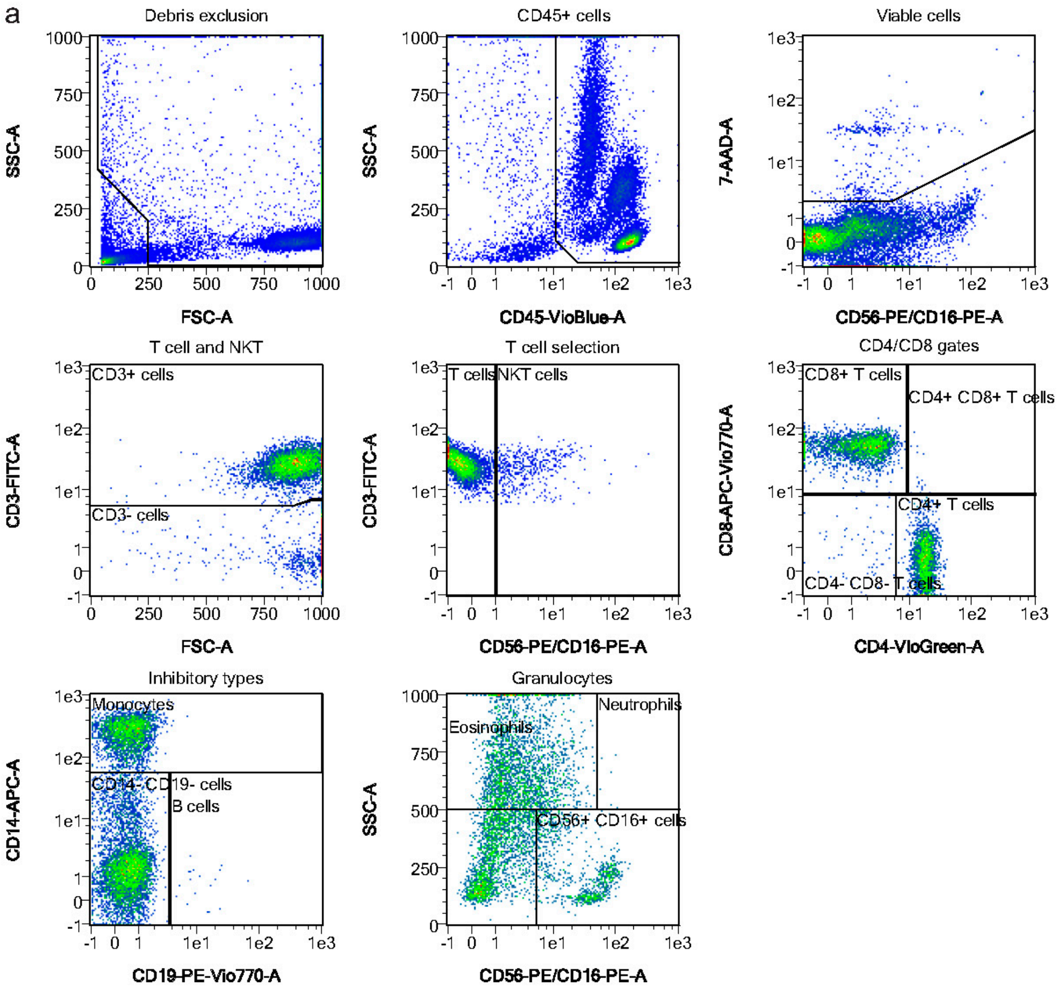

b

Cell type	Defined population	Cells/mL	Cells/mL x 1.0	Count	[%] in viable CD45+ cells
Sample	all acquired events	1.18E+05	1.18E+05	23453	
Debris exclusion	FSC small events excluded	7.99E+04	7.99E+04	15903	
All viable cells	FSC small events excluded and 7-AAD-	7.77E+04	7.77E+04	15462	
CD45+ cells	FSC small events excluded and CD45+	7.13E+04	7.13E+04	14197	
Viable CD45+ cells	CD45+ 7-AAD-	6.94E+04	6.94E+04	13809	
CD3+ cells	Viable CD3+	2.78E+04	2.78E+04	5532	40.06
CD3- cells	Viable CD3-	4.16E+04	4.16E+04	8277	59.94
T cells	Viable CD3+ CD56- CD16-	2.55E+04	2.55E+04	5075	36.75
NKT cells	Viable CD3+ CD56+ CD16+	2.30E+03	2.30E+03	457	3.31
CD4+ cells	Viable CD3+ CD56- CD16- CD4+ CD8-	1.17E+04	1.17E+04	2332	16.89
CD8+ cells	Viable CD3+ CD56- CD16- CD4- CD8+	1.34E+04	1.34E+04	2665	19.30
CD4+ CD8+ cells	Viable CD3+ CD56- CD16- CD4+ CD8+	8.54E+01	8.54E+01	17	0.12
Monocytes	Viable CD3- CD14+	1.46E+04	1.46E+04	2900	21.00
B cells	Viable CD3- CD19+	9.05E+01	9.05E+01	18	0.13
Neutrophils	Viable CD3- CD14- CD19- CD56+ CD16+ SSC hi	3.02E+01	3.02E+01	6	0.04
Eosinophils	Viable CD3- CD14- CD19- CD56- CD16- SSC hi	1.22E+04	1.22E+04	2419	17.52
CD56+ CD16+ cells	Viable CD3- CD14- CD19- CD56+ CD16+ SSC lo	4.35E+03	4.35E+03	866	6.27
					[%-#]
Viability of CD3+ cells	CD45+ CD3+ 7-AAD- among CD45+ CD3+	2.78E+04	2.78E+04	5532	97.67
% CD3+ in viable CD45+	CD45+ 7-AAD- CD3+ among CD45+ 7-AAD-	2.78E+04	2.78E+04	5532	40.06

Panel (a) Dot plots for immunophenotyping of immune cell subsets in the starting cell material (product of apheresis).

Panel (b) Quantification of cell populations (e.g., CD3+ T cells, CD19+ B cells, CD56+ NK cells, monocytes, etc.) in the apheresis product.

Figure S6. Representative analysis of CAR-T cell product manufactured using the CliniMACS Prodigy system

a

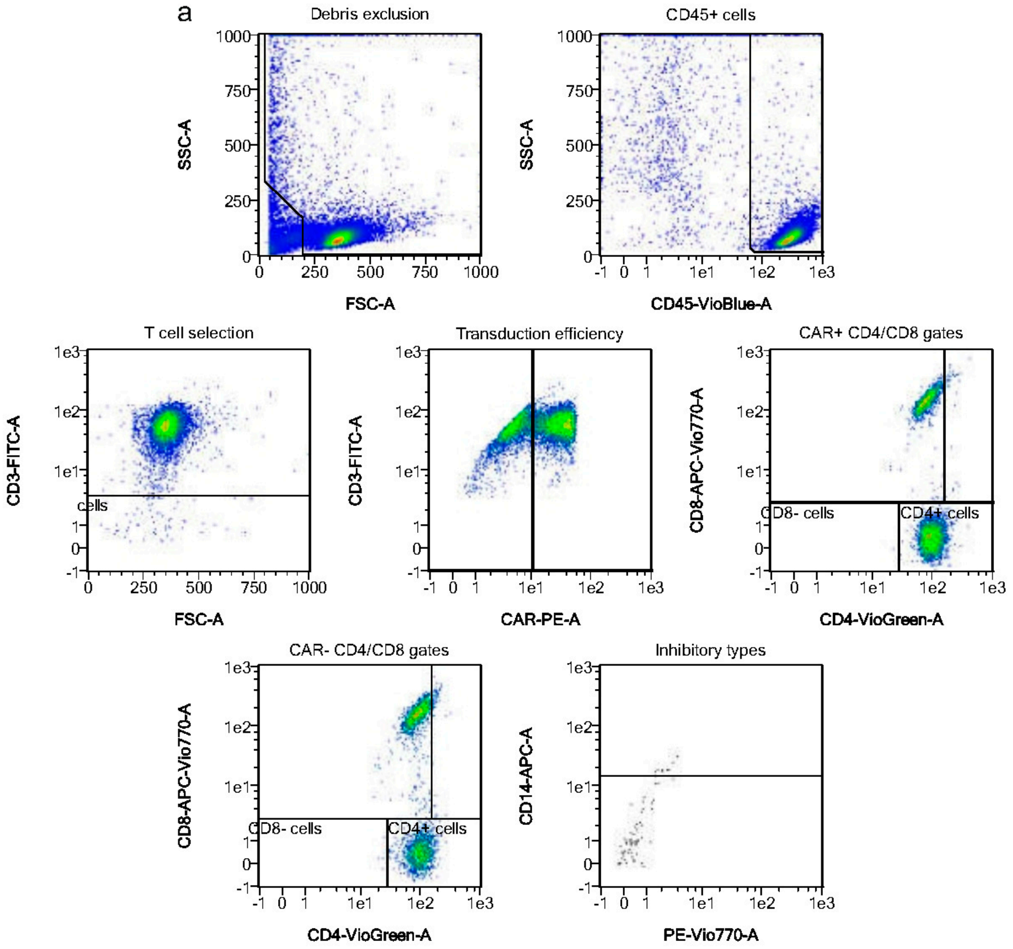

b

Cell type	Defined population	Cells/mL	Cells/mL x 1.0	Count	[%-#]
Sample	all acquired events	5.84E+05	5.84E+05	28689	
Debris exclusion	FSC small events excluded	5.04E+05	5.04E+05	24758	86.30
CD45+ cells	FSC small events excluded and CD45+	4.78E+05	4.78E+05	23494	94.89
Viable CD45+ cells	CD45+ 7-AAD-	1.32E+05	1.32E+05	6486	27.61
CD3+ cells	Viable CD3+	1.31E+05	1.31E+05	6421	99.00
CAR+ cells	Viable CD3+ CAR+	7.50E+04	7.50E+04	3686	57.40
CAR+ CD4+ cells	Viable CD3+ CAR+ CD4+ CD8-	3.16E+04	3.16E+04	1552	42.10
CAR+ CD8+ cells	Viable CD3+ CAR+ CD4- CD8+	3.78E+04	3.78E+04	1858	50.40
CAR+ CD4+ CD8+ cells	Viable CD3+ CAR+ CD4+ CD8+	6.52E+02	6.52E+02	32	0.87
CAR+ CD4- CD8- cells	Viable CD3+ CAR+ CD4- CD8-	4.97E+03	4.97E+03	244	6.63
CAR- cells	Viable CD3+ CAR-	5.57E+04	5.57E+04	2735	42.60
CAR- CD4+ cells	Viable CD3+ CAR- CD4+ CD8-	4.70E+04	4.70E+04	2306	84.30
CAR- CD8+ cells	Viable CD3+ CAR- CD4- CD8+	8.18E+03	8.18E+03	402	14.70
CAR- CD4+ CD8+ cells	Viable CD3+ CAR- CD4+ CD8+	4.48E+02	4.48E+02	22	0.79
CAR- CD4- CD8- cells	Viable CD3+ CAR- CD4- CD8-	1.22E+02	1.22E+02	6	0.21
CD3- cells	Viable CD3-	9.16E+02	9.16E+02	45	0.70
Monocytes	Viable CD3- CD14+ among CD45+ 7-AAD-	1.83E+02	1.83E+02	9	0.14
					[%]
CAR+ cells in viable CD3+	CD3+ CAR+ in CD45+ 7-AAD- CD3+	7.50E+04	7.50E+04	3686	57.40
CAR+ cells in viable cells	CD3+ CAR+ in CD45+ 7-AAD-	7.50E+04	7.50E+04	3686	56.82

Panel (a) Dot plots for immunophenotyping of CAR-T cell, non-transduced T cell and residual immune cell subsets (e.g., B cells, NK cells, monocytes) in CAR-T product (#4).
Panel (b) Quantification of CAR-T cell (CAR+), non-transduced T cell (CAR-), and admixing cell type frequencies in CAR-T product (#4).

Table S2. Viability, cell counts and phenotyping of T cells in manufactured anti-CD19 cell products [1]

Product #	1	2	3	4	5	6	7	8	9	10	11	12
CAR used	CAR.TM8-BBz						CAR.TM28-28z					
Viability at end of process (Day 12), %	93.8	95.3	96.2	95.2	95.7	93.3	94.9	95.7	94.9	95.4	96.8	95.6
Total cells (Day 1)	1.00E+08	1.00E+08	1.00E+08	1.00E+08	1.00E+08	1.00E+08	1.00E+08	1.00E+08	1.00E+08	1.00E+08	1.00E+08	1.00E+08
Total cells (Day 3)	1.58E+08	2.09E+08	2.03E+08	1.54E+08	2.70E+08	1.82E+08	1.72E+08	2.43E+08	1.56E+08	2.15E+08	1.58E+08	2.09E+08
Total cells (Day 4)							1.98E+08	3.36E+08	3.11E+08	4.14E+08	1.91E+08	3.41E+08
Total cells (Day 5)	3.07E+08	4.27E+08	6.80E+08	2.28E+08	7.58E+08	6.03E+08	2.56E+08	5.47E+08	3.41E+08	5.79E+08	3.98E+08	5.98E+08
Total cells (Day 6)							3.74E+08	8.84E+08	4.05E+08	1.11E+09	6.16E+08	8.77E+08
Total cells (Day 7)	4.95E+08	8.71E+08	1.39E+09	4.31E+08	1.79E+09	1.38E+09	6.34E+08	1.20E+09	5.80E+08	1.54E+09	9.55E+08	1.24E+09
Total cells (Day 10)	7.00E+08	1.26E+09	2.49E+09	5.28E+08	3.27E+09	2.00E+09	8.67E+08	2.04E+09	8.53E+08	2.63E+09	1.70E+09	1.71E+09
Total cells (Day 12)	8.55E+08	1.86E+09	3.29E+09	6.09E+08	3.75E+09	2.57E+09	1.16E+09	3.23E+09	1.05E+09	3.25E+09	2.24E+09	2.60E+09
Activation Markers on Day 2												
CD25+ (% of CD3+)	84.9	87.5	91.5	90.3	84.3	93.5	75.9	87.3	87.9	79.8	90.2	91.9
CD69+ CD25+ (% of CD25+)	23.3	19.6	18.9	34.1	52.1	37.7	21.9	45.9	11.1	21.9	49.3	39.7
CD69- CD25+ (% of CD25+	76.7	80.4	81.1	65.9	47.9	62.3	78.1	54.1	88.9	78.1	50.7	60.3

)												
Transduction efficiency												
Percent of CAR+ cells	39.3	48.2	43.8	57.4	48.7	44.8	40.6	45.8	32.8	31.6	41.9	51.4
Phenotyping of T cell subsets												
CD4+CAR+ (% of CAR+)	49.7	64	70.2	42.1	63.6	65.4	63.1	81.6	67	82.7	53.4	58.4
CD8+CAR+ (% of CAR+)	50.1	33.9	28.4	50.4	34.2	33.3	28.9	16.2	29.2	16.8	40.9	38.3
CD4+CAR- (% of CAR-)	46.8	67	77.4	84.3	67.9	50.9	68.9	59.7	66.2	51.3	58.7	45.1
CD8+CAR- (% of CAR-)	52.4	31.4	19.2	14.7	30.9	40.9	29.4	38.3	31.8	46.8	40.2	51.6
CD3-	0.6	0.8	0.6	0.7	0.6	0.7	0.7	0.7	0.9	0.7	0.8	0.6
T-cell Subsets (% of CAR+ population)												
Naïve (Tn) CD4+CAR+ (% of CD4+CAR+)	<0.1	<0.1	<0.1	<0.1	<0.1	<0.1	<0.1	<0.1	<0.1	<0.1	<0.1	<0.1
Central memory (Tcm) CD4+CAR+ (% of CD4+CAR+)	71.6	53.8	85.4	44.3	62.3	65.1	43.8	62.8	57.9	42.2	43.3	50.1
Effector memory (Tem) CD4+CAR+ (% of CD4+CAR+)	27.8	45.2	13.4	55.2	37.1	34.1	55.3	36.6	41.6	57.3	56.1	49.4

Temra CD4+CAR+ (% of CD4+CAR+)	0.5	0.9	1.1	0.4	0.5	0.7	0.8	0.5	0.4	0.4	0.5	0.4
Naïve (Tn) CD8+CAR+ (% of CD8+CAR+)	<0.1	<0.1	<0.1	<0.1	<0.1	<0.1	<0.1	<0.1	<0.1	<0.1	<0.1	<0.1
Central memory (Tcm) CD8+CAR+ (% of CD8+CAR+)	67.1	56.7	57.3	48.9	51.1	68.9	26.4	23.2	32.9	38.8	20.1	37.9
Effector memory (Tem) CD8+CAR+ (% of CD8+CAR+)	32.7	43.1	42.5	50.9	48.6	30.9	72.5	76.6	66.2	59.7	79.8	61.7
Temra CD8+CAR+ (% of CD8+CAR+)	0.1	0.1	0.1	0.1	0.2	0.1	1	0.1	0.8	1.4	0	0.3

[1] T-cell subsets were defined as: naïve (Tn, CD45RA+CCR7+), central memory (Tcm, CD45RA-CCR7+), effector memory (Tem, CD45RA-CCR7-), and terminally differentiated effector memory RA-expressing (Temra, CD45RA+CCR7-). Staining protocol and antibody panels are described in the article. Gating strategies are detailed in Supplementary Figures S1–S4.

Table S3. Cytokine production in cocultures of CAR+ cells with B cells [1]

Product #	1	2	3	4	5	6	7	8	9	10	11	12
IFN-γ, pg/ml												
CAR$^+$ T cells co-cultured with B cells	392	214	258	344	230	184	378	520	390	360	444	662
NT cells with B cells	0	0	0	0	0	0	0	0	0	0	0	0
CAR$^+$ T cells without target cells	0	0	0	0	0	0	5	0	0	0	6	5
TNF-α, pg/ml												
CAR$^+$ T cells co-cultured with B cells	58	43	43	49	30	21	74	131	81	91	90	182
NT cells with B cells	0	0	0	0	0	0	0	0	0	0	0	0
CAR$^+$ T cells without targets	0	0	0	0	0	0	0	3	0	0	0	5
IL-2, pg/ml												
CAR-T cells with B cells	39	0	29	19	32	17	93	122	100	43	117	119
NT cells with B cells	0	0	0	0	0	0	0	0	0	0	0	0
CAR-T cells without targets	0	0	0	0	0	0	0	0	0	0	0	0

[1] NT, Non-transduced T cells

Table S4. Cytotoxicity of CAR+ cells in cocultures with B cells [1]

Product #	1	2	3	4	5	6	7	8	9	10	11	12
Percent of specific killing												
E:T (10:1)	77	33	47	69	36	25	36	65	45	36	60	89
E:T (5:1)	65	25	38	52	26	17	22	46	32	26	44	59
NT	1.7	2.4	0.9	3.2	1.2	1.9	1.2	2.5	3.1	1.4	2.8	1.4

[1] E:T, Effector (CAR+) / Target (B cell) ratio
NT, Non-transduced T cells
